# Repeat Influenza Vaccination Effects in 2021/22 and 2022/23 in a Community-Based Cohort in Hong Kong

**DOI:** 10.1093/infdis/jiag051

**Published:** 2026-01-28

**Authors:** Jennifer C Zhong, Shuyi Zhong, Lisa Touyon, Faith Ho, Niki Y M Au, Samuel M S Cheng, Dennis K M Ip, Malik Peiris, Emily T Martin, Sarah Cobey, Sook-San Wong, Nancy H L Leung, Benjamin J Cowling

**Affiliations:** WHO Collaborating Centre for Infectious Disease Epidemiology and Control, School of Public Health, Li Ka Shing Faculty of Medicine, The University of Hong Kong, Pokfulam, Hong Kong Special Administrative Region, China; WHO Collaborating Centre for Infectious Disease Epidemiology and Control, School of Public Health, Li Ka Shing Faculty of Medicine, The University of Hong Kong, Pokfulam, Hong Kong Special Administrative Region, China; Pasteur Research Pole, School of Public Health, LKS Faculty of Medicine, The University of Hong Kong, Pokfulam, Hong Kong Special Administrative Region, China; WHO Collaborating Centre for Infectious Disease Epidemiology and Control, School of Public Health, Li Ka Shing Faculty of Medicine, The University of Hong Kong, Pokfulam, Hong Kong Special Administrative Region, China; WHO Collaborating Centre for Infectious Disease Epidemiology and Control, School of Public Health, Li Ka Shing Faculty of Medicine, The University of Hong Kong, Pokfulam, Hong Kong Special Administrative Region, China; WHO Collaborating Centre for Infectious Disease Epidemiology and Control, School of Public Health, Li Ka Shing Faculty of Medicine, The University of Hong Kong, Pokfulam, Hong Kong Special Administrative Region, China; WHO Collaborating Centre for Infectious Disease Epidemiology and Control, School of Public Health, Li Ka Shing Faculty of Medicine, The University of Hong Kong, Pokfulam, Hong Kong Special Administrative Region, China; WHO Collaborating Centre for Infectious Disease Epidemiology and Control, School of Public Health, Li Ka Shing Faculty of Medicine, The University of Hong Kong, Pokfulam, Hong Kong Special Administrative Region, China; Centre for Immunology & Infection (C2i), Hong Kong Science and Technology Park, New Territories, Hong Kong Special Administrative Region, China; Department of Epidemiology, School of Public Health, University of Michigan, Ann Arbor, Michigan, USA; Department of Ecology and Evolution, University of Chicago, Chicago, Illinois, USA; WHO Collaborating Centre for Infectious Disease Epidemiology and Control, School of Public Health, Li Ka Shing Faculty of Medicine, The University of Hong Kong, Pokfulam, Hong Kong Special Administrative Region, China; Centre for Immunology & Infection (C2i), Hong Kong Science and Technology Park, New Territories, Hong Kong Special Administrative Region, China; WHO Collaborating Centre for Infectious Disease Epidemiology and Control, School of Public Health, Li Ka Shing Faculty of Medicine, The University of Hong Kong, Pokfulam, Hong Kong Special Administrative Region, China; WHO Collaborating Centre for Infectious Disease Epidemiology and Control, School of Public Health, Li Ka Shing Faculty of Medicine, The University of Hong Kong, Pokfulam, Hong Kong Special Administrative Region, China

**Keywords:** influenza, vaccination, antibody, hemagglutination inhibition

## Abstract

**Background:**

Repeated influenza vaccination has been associated with attenuated immune responses and reduced clinical effectiveness. Most studies have focused on the responses at 30 days post-vaccination. As influenza did not circulate in Hong Kong during the COVID-19 pandemic, we were able to investigate vaccine responses in the absence of infections.

**Methods:**

In this community-based cohort study in Hong Kong, we investigated the impact of repeated annual influenza vaccination on antibody titer boosting and waning rates of 8 contemporary vaccine strains in 2021/22 and 2022/23, using hemagglutination inhibition assays. We estimated the impact of repeat vaccination on mean fold rises and on post-vaccination titers. We investigated differences by vaccination history in titer waning rates using a power law model.

**Results:**

We found differences in post-vaccination geometric mean and mean fold rises in titers against several vaccine strains in repeat vaccinees in 2022/23 compared to 2021/22. However, although participants with higher vaccination uptake had significantly slower antibody waning against A/Victoria/2570/2019, B/Phuket/3073/2013, and B/Austria/1359417/2019, they reached similar antibody titers at 6 months post-vaccination.

**Conclusions:**

Our findings suggest that repeated influenza vaccination is associated with reduced titer increase at day 14 post-vaccination, but has less impact on antibody levels at 6 months post-vaccination.

## Teaser

This study in Hong Kong, during the COVID-19 pandemic with no influenza circulation, showed repeated annual influenza vaccination is associated with attenuated antibody boosts post-vaccination but similar titers at 6 months compared to individuals with less frequent vaccination history.

Annual vaccinations against influenza have been recommended since the 1960s to reduce the public health impact of influenza epidemics [1]. The strains included in influenza vaccines are regularly updated to keep up with antigenic drift in circulating strains [2]. Although annual vaccination has been shown to be the best protective measure against influenza, a growing body of evidence has reported that recipients of repeated influenza vaccination can experience attenuated antibody responses compared to first-time vaccinees in some years, albeit repeat vaccinees tend to possess higher baseline titers [3–9]. These reduced antibody responses were thought to be associated with reduced vaccine effectiveness [5]. Furthermore, it has been shown that both pre-vaccination titer levels and boosting levels can affect the waning rate [10, 11]. This highlights the need for a deeper understanding of how repeated influenza vaccination shapes antibody responses and long-term protection.

During the COVID-19 pandemic in Hong Kong, there was no influenza transmission for three years between March 2020 and February 2023 [12], providing an opportunity to explore antibody dynamics after influenza vaccination in a population without any infections. Previous studies of repeated vaccination effects are often limited to only 1 season [5, 13–15], and immunogenicity studies often focus on the post-vaccination antibody responses at a single time point after vaccination. We have established a cohort of community-dwelling individuals in Hong Kong, and here, we examined the effects of repeated influenza vaccination on antibody responses and waning in the 2021/22 and the 2022/23 seasons.

## METHODS

### Participants

We analyzed data from the “Evaluating Population Immunity in Hong Kong” (EPI-HK) cohort, an ongoing community-based sero-epidemiological study in Hong Kong [16]. A total of 2663 participants were enrolled beginning June of 2020, and follow-up continues to present. EPI-HK recruited participants of any age currently residing in Hong Kong and planning to reside there for at least the following year. Participants over the age of 6 at recruitment were asked to provide blood samples at least 2 times during the study. Those showing signs of dementia or significant cognitive impairment and unable to give consent were excluded from the study.

The study uses an open cohort design, allowing for new recruitment every year to replace participants who withdraw from the study. Each year, participants provide updates on demographic information, medical history, and influenza vaccination status. At enrollment to the cohort, we record influenza vaccination history for the prior 6 years. Participants provide blood samples approximately every 6 months. Additional blood samples are collected within 7 days before and between 21 and 42 days after influenza and SARS-CoV-2 vaccinations where possible. Participants receive coupons worth HK$100 (approximately US$13) for each blood draw. We conduct active surveillance for respiratory illness, with collection of swabs for PCR testing, but paused surveillance during the prolonged period when there was no community circulation of influenza in Hong Kong that encompasses the years studied here [12].

### Ethics

Written informed consent was obtained from all participants, with parental consent for children < 18 years of age. The study protocol was approved by the Institutional Review Board of the University of Hong Kong.

### Laboratory Analysis

A subset of participants aged ≥18 years who reported receipt of inactivated influenza vaccine in 2021/22 as well as in 2022/23 and provided at least 4 serum samples during the corresponding period were selected. Sera were extracted from clotted blood samples within 24 h after collection, divided into 2–4 aliquots, and stored at −80°C until subsequent serologic testing. Serum samples were tested by hemagglutination inhibition (HAI) assay in serial 2-fold dilutions starting at 1:10 to a maximum dilution of 1:1280 as previously described [17]. Titers < 10 were coded as 5, while those ≥1280 were coded as 1280. Sera were tested for the presence of antibodies against the 2021/22 and 2022/23 vaccine strains that the participants received for each respective seasons: A/Victoria/2570/2019 (H1N1), A/Cambodia/e0826360/2020 (H3N2), A/Darwin/9/2021 (H3N2), B/Phuket/3073/2013 (Yamagata lineage), B/Washington/02/2019 (Victoria lineage), and B/Austria/1359417/2021 (Victoria lineage) (see Supplementary Table 1). Sera were also tested against 2 vaccine strains from prior influenza seasons: A/Brisbane/2/2018 (H1N1) (2019/20) and A/Hong Kong/4801/2014 (H3N2) (2016/17–2017/18). These 2 strains were also selected to represent the strains circulating in Hong Kong for several years prior to the COVID-19 pandemic. Sera from the same participant were tested in parallel. HAI antibody titers were read as the reciprocal of the highest serum dilution causing complete inhibition of agglutination.

### Statistical Analysis

It is well established that individuals with higher pre-vaccination antibody titers tend on average to have reduced mean fold rises (MFRs) from pre-vaccination to post-vaccination, and this is often described as the “antibody ceiling effect” [4, 18, 19]. Repeat influenza vaccination effects are identified as potentially reduced MFR in antibody titers from pre-vaccination to post-vaccination which occur beyond what would be predicted by ceiling effects [10, 20]. Repeat vaccination effects can also be identified as potentially reduced post-vaccination geometric mean titers (GMTs) against vaccine strains in repeat vaccinees versus first-time vaccinees [6, 7, 21, 22]. We therefore investigated the potential occurrence of repeat vaccination effects in MFRs and in post-vaccination GMTs.

We calculated the GMTs and MFRs from before vaccination to up to 60 days after influenza vaccination, in 2021/22 and then in 2022/23. We tested for a difference in MFR and the logarithm of the post-vaccination titers (GMTs) between 2021/22 and 2022/23 using an analysis of variance (ANOVA) test and a Mann–Whitney U test, respectively. We used a power law model represented by the curve log(*y*) = α + β log(*x*-14) to quantify antibody waning kinetics in each participant starting from the presumed peak in titer at approximately 14 days following 2021/22 vaccination, where *y* is the HAI titer, α is the intercept, β is the slope of the model, and *x* is the time in days post-vaccination [23]. We included all available data prior to receipt of 2022/23 vaccination. Parameters were estimated by fitting a linear mixed effects model. To account for person-to-person variation in vaccine response, the intercept α was modeled as a random effect for each individual, with a common slope β for each vaccination history group. Alternative model formulations were examined in sensitivity analyses (See Supplementary Table 2). An average waning curve for each vaccination history group was constructed by taking the geometric mean of the intercept of all of the individually fitted curves in that group and the common slope. The association between waning rate and number of prior vaccinations in the preceding 6 years was assessed by log-linear regression, adjusted for age and sex. Participants, all of whom were vaccinated in 2021/22 and 2022/23, were divided into three groups based on vaccination history in the 6 preceding years: no recent history (0 prior vaccinations), low uptake (1–2 prior vaccinations), and high uptake (more than 3 prior vaccinations). HAI titers at 14 days and 180 days post-vaccination were estimated from the fitted waning models. All analyses were carried out using R version 4.4.1 (R Foundation for Statistical Computing, Vienna, Austria).

## RESULTS

**Figure 1.**
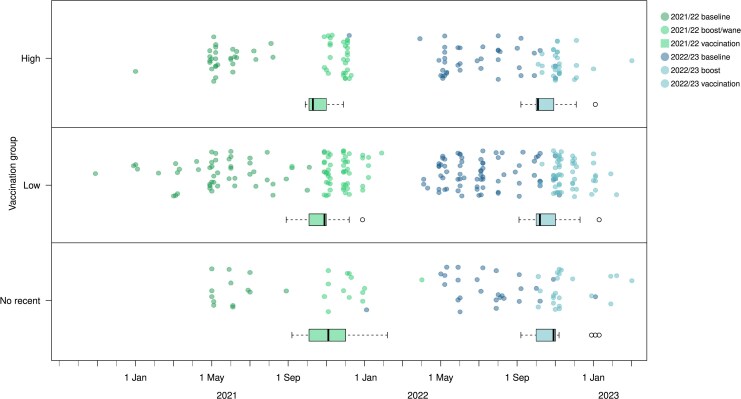
Sera included in this analysis were collected from the end of 2020 though to early 2023. All participants were vaccinated in both the 2021/22 and the 2022/23 seasons. Our analysis of antibody waning following vaccination in 2021/22 included relevant data points prior to vaccination in 2022/23. Box and whisker plots indicate the distribution of times of vaccination in each year and vaccination history group, where no recent vaccination, low and high uptake represent 0, 1–2, and 3+ vaccinations in the past 6 years, respectively.

In the EPI-HK cohort, we enrolled a total of 2663 participants starting from June 2020, with follow-up ongoing in 2025. For the current analyses, we selected 180 participants aged ≥18 years who reported receipt of inactivated influenza vaccine in 2021/22 as well as in 2022/23 and met at least one of the following criteria: one pre-vaccination sample and one sample between 14 and 60 days post-vaccination for either year for post-vaccination boosting analysis, or one pre-2021/22 vaccination sample and 2–3 samples between 14 days post-2021/22 vaccination up to 2022/23 vaccination for the analysis of antibody waning dynamics. In total, we identified 554 serum samples for analysis. Vaccination and serum sample collection times are described in Figure 1. Tested strains and vaccine compositions in the 2 years are reported in Supplementary Table 1.

To assess immune response to vaccination, we measured antibody titers using HAI assays in 142 participants who provided a serum sample within 60 days after vaccination. Ninety-six participants were included in 2021/22 and 107 in 2022/23 based on serum sample collection times, with 61 participants included in both years. To assess antibody waning dynamics after vaccination, 171 participants who provided at least 2 serum samples between 14 days post-2021/22 vaccination and 60 days before 2022/23 vaccination were selected. Demographic characteristics and number of prior vaccinations in the preceding 6 years of the 180 participants are shown in Table 1. Most participants reported also receiving at least one influenza vaccination in the preceding 6 years (86.5% in 2021/22, 78.5% in 2022/23), and there were no statistically significant differences in post-vaccination serum collection timing between vaccination history groups.

**Table 1.** Selected Characteristics of 180 Participants, Summarized by Immunization Year and Analysis of Responses to 2021/22 Vaccination, 2022/23 Vaccination, or Antibody Waning Following Receipt of 2021/22 Vaccination

Characteristic	2021/22 Vaccination (n = 96)		2022/23 Vaccination (n = 107)		2021/22 Waning (n = 171)	
	No.	%	No.	%	No.	%
Age group, years						
18–29	13	13.5	13	12.1	27	15.8
30–44	25	26.0	18	16.8	37	21.6
45–54	21	21.9	32	30.0	37	21.6
55–64	23	24.0	27	25.2	33	19.3
≥65	14	14.6	17	15.9	37	21.6
Female sex	47	48.9	51	47.6	84	49.1
Number of prior influenza vaccinations from 2015/16 to 2020/21						
None	13	13.5	23	21.5	34	19.9
1–2	52	54.2	53	49.5	81	47.4
3 or more	31	32.3	31	29.0	56	32.7

**Figure 2.**
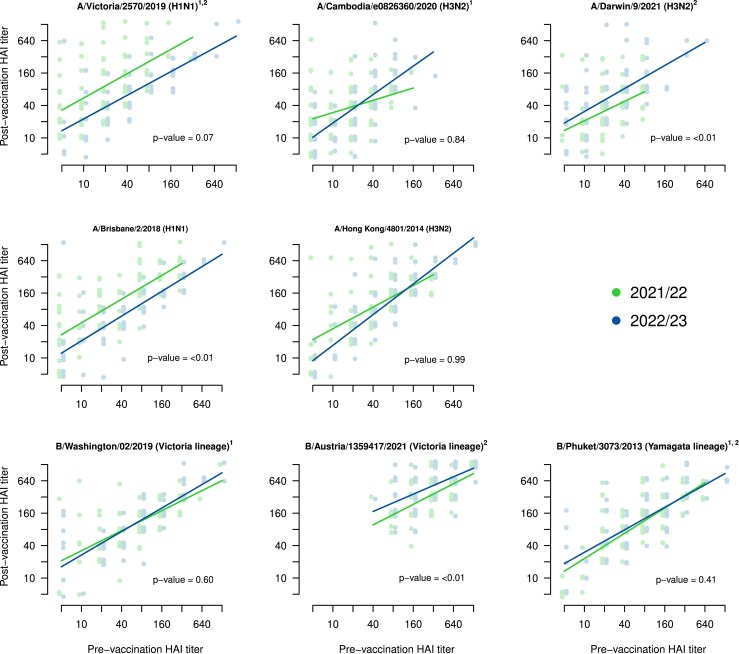
Observed post-vaccination hemagglutination inhibition (HAI) antibody titers after 14–60 days by pre-vaccination titers against 8 influenza vaccine strains. Points represent titers in 2021/22 and 2022/23 with lines representing the average geometric mean titers (GMT) by pre-vaccination titer. A lower average GMT observed in 2022/23 is indicative of ceiling effect; *P* values shown in each panel reflect the Mann–Whitney U test for a statistically significant difference in GMT between the 2 groups. Superscripts indicate whether the strain was included in the northern hemisphere influenza vaccine for 2021/22 (1) and/or 2022/23 (2).

Figure 2 shows the post-vaccination titers after vaccination in both seasons, with a regression line to show the average post-vaccination titer by pre-vaccination HAI titer level. Lower post-vaccination titers were observed against A/Victoria/2570/2019 (H1N1) (NH vaccine in both years) in 2022/23 than in 2021/22. For A/Cambodia/e0826360/2020(H3N2) (2021/22 vaccine), post-vaccination HAI titers were higher in 2021/22 compared to 2022/23 for those with low baseline titers but lower for those with high baseline titers. In contrast, A/Darwin/9/2021(H3N2) (2022/23 vaccine) had statistically significantly higher post-vaccination titers in 2022/23 compared to 2021/22. Post-vaccination HAI titers against A/Brisbane/2/2018(H1N1) (2019/20 vaccine) were statistically significantly higher in 2021/22 than 2022/23 for all pre-vaccination titer levels. For A/Hong Kong/4801/2014(H3N2) (the 2016/17-2017/18 vaccine), post-vaccination HAI titers were higher in 2021/22 compared to 2022/23 for low baseline titers but lower for high baseline titers. Finally, all B lineage strains had higher post-vaccination titers in 2022/23 compared to 2021/22, with B/Austria/135941/2021(Victoria lineage) (2022/23 vaccine) being statistically significant.

**Figure 3.**
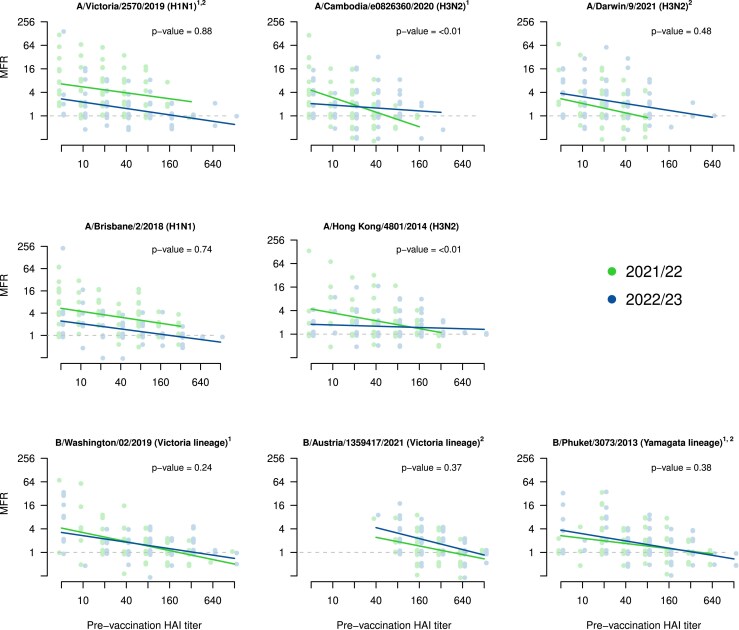
Observed mean fold rise (MFR) after 14–60 days in hemagglutination inhibition (HAI) antibody titers by pre-vaccination titers against 8 contemporary influenza strains. Points represent titers in 2021/22 and 2022/23 with the lines representing the average MFR by pre-vaccination titer. A gray dashed line shows an MFR of 1, or no change post-vaccination. Vertical dashed lines show the geometric mean pre-vaccination titers for 2021/22 and 2022/23. A lower average MFR observed in 2022/23 is indicative of vaccine attenuation, *P* values shown in each panel reflect the ANOVA test for a difference in slopes (ie, mean fold rise) between the 2 groups after taking ceiling effects into account. Superscripts indicate whether the strain was included in the northern hemisphere influenza vaccine for 2021/22 (1) and/or 2022/23 (2).

Figure 3 shows the MFR in 8 contemporary strains after vaccination in both seasons with fitted regression lines to show the average MFR by pre-vaccination HAI titer level. The geometric mean of the pre-vaccination titer was higher in 2021/22 than 2022/23 for all strains save for B/Austria/135941/2021(Victoria lineage). In all vaccine strains, a greater range in MFR was observed in individuals with lower pre-vaccination titer levels compared to higher pre-vaccination titer levels. There was a statistically significant difference in MFR by pre-vaccination titers for A/Hong Kong/4801/2014(H3N2) and A/Cambodia/e0826360/2020(H3N2) in 2021/22 versus 2022/23.

**Figure 4.**
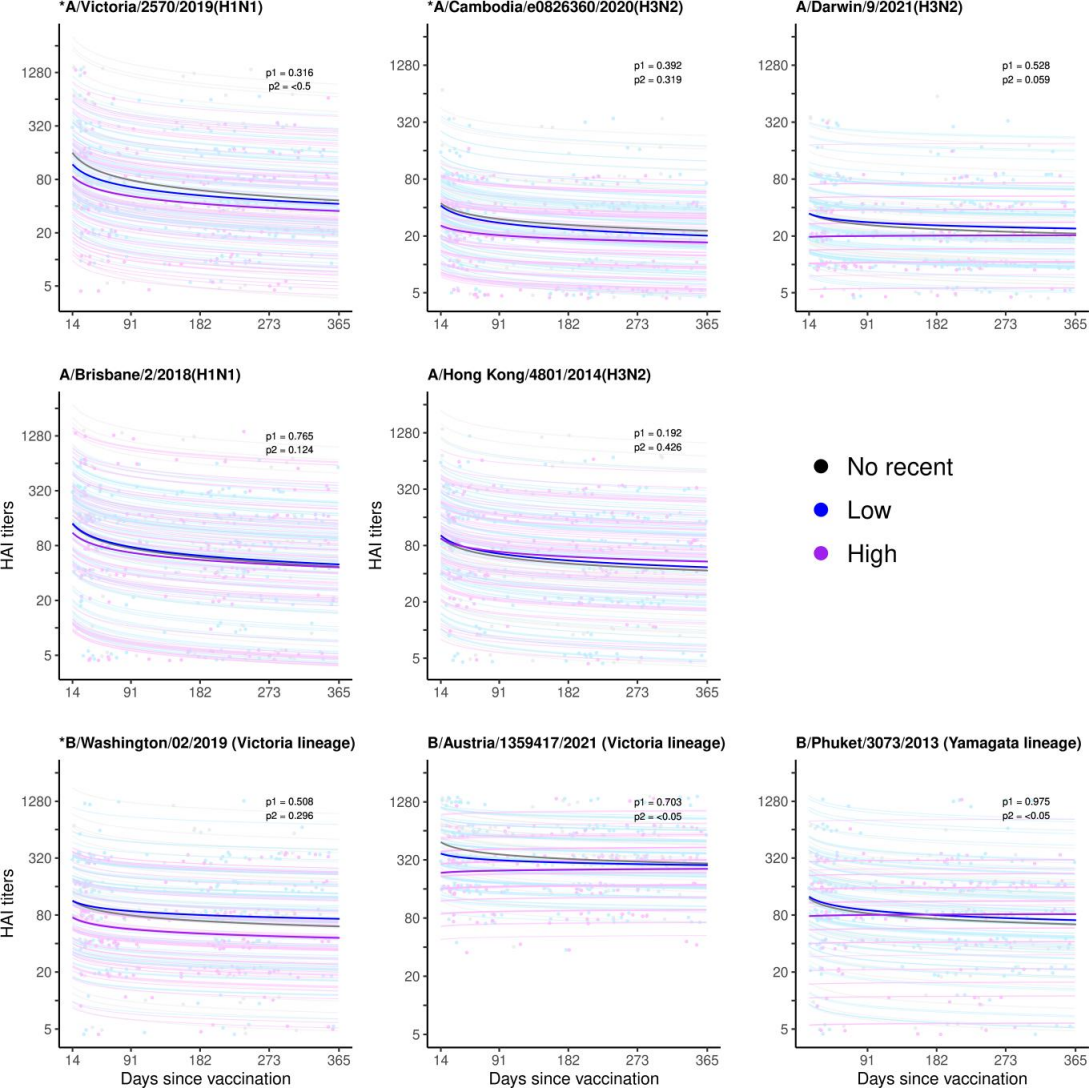
Estimated patterns in waning of hemagglutination inhibition (HAI) antibody titers from 14 days to 365 days following receipt of 2021/22 influenza vaccination using power law models, stratified by vaccine history. Black curves represent the group with no recent vaccinations (0 prior vaccinations), blue represents low vaccine uptake (1–2 prior vaccinations), and purple represents high vaccine uptake (3+ prior vaccinations) from 2014/15 to 2020/21. The *P* values in each panel (p1 and p2) reflect the multiple linear regression test for a difference in waning rate between the group with no recent vaccinations and the low and high vaccination groups respectively. * indicates strains in the 2021/22 northern hemisphere influenza vaccine.

Prior vaccination history was not associated with the timing of vaccination or return visit for blood draw in 2021/22 and 2022/23. On average, repeat vaccinees were vaccinated around the same time in 2021/22 as the group who had no recent vaccinations, with more than half of vaccinations received in the months of October and November each year (Figure 1). An average waning curve post 2021/22 vaccination was constructed for each vaccination history group and the waning rates compared (Figure 4). The estimated HAI titer at 14 days post-vaccination was lower in the high uptake group than the group with no recent prior vaccination for all vaccine strains. The estimated HAI titers at 180 days post-vaccination for both vaccination groups were within 2-fold differences of the group with no recent vaccinations for all strains (Supplementary Table 3). Of note, HAI titers were substantially lower against A/Cambodia/e0826360/2020(H3N2) than the other 2021/22 vaccine strains, with all day 14 and day 180 estimates close to or below the HAI antibody titer threshold of 40, particularly in the higher uptake group (Supplementary Table 3). A statistically significant positive association was found between waning rate and A/Victoria/2570/2019(H1N1), B/Austria/1349517/2021(Victoria lineage), and B/Phuket/3073/2013(Yamagata lineage) in the higher vaccination uptake group (Figure 4). No statistically significant associations were found for the lower uptake group.

## DISCUSSION

In this study of 180 participants from a community-based cohort in Hong Kong during the 2021/22 and 2022/23 seasons, we found that repeat vaccinees had higher pre-vaccination titers, weaker antibody boosts after vaccination, but slower antibody waning to reach similar antibody titers after 6 months compared to first-time vaccinees. Reduced HAI titer responses in repeat vaccinees have been previously reported [5, 24–26] and were observed here (Figure 2). We found statistically significantly higher post-vaccination titers in 2022/21 versus 2022/23 for A/Brisbane/2/2018, a strain from a past vaccine season, suggesting that cross-reactive antibody boosting may decrease over time. A similar difference was seen for A/Victoria/2570/2019(H1N1), a vaccine strain used in both seasons of the study. Antibody dynamics following vaccination have been described in previous studies [27–29] but could have been affected by influenza circulation. Here, we found a statistically significantly slower waning in the group with ≥3 prior vaccinations for A/Victoria/2570/2019 and B/Phuket/3073/2013 compared to the other 2 groups with less vaccination history. Although repeat vaccinees had lower estimated antibody titers at 14 days post-vaccination, all strains waned to within 2-fold differences by 180 days post-vaccination, indicating that initial differences in post-vaccination antibody titers were more substantial than longer-term differences between the groups with different numbers of prior vaccinations.

We observed weaker antibody boosting (MFRs) in all influenza A strains except for A/Darwin/9/2021(H3N2) in 2022/23 versus 2021/22 (Figure 2), which is consistent with previously reported findings on the ceiling effect [6, 30]. Notably, A/Darwin/9/2021(H3N2) was part of the 2022/23 northern hemisphere vaccine while the others were all included in the 2021/22 vaccine or earlier. This demonstrates that even a single repeated vaccination can have noticeable effects, but there may be a less degree of attenuation when the vaccine strain is updated, consistent with the antigenic distance hypothesis [31]. When accounting for the ceiling effect by using the MFR, we found a statistically significant difference between 2021/22 and 2022/23 for A/Hong Kong/4801/2014(H3N2) and A/Cambodia/e0826360/2020(H3N2), indicating that attenuation in vaccine effectiveness may be mediated by cross-reactivity conferred by repeat vaccinations. We observed that participants had higher post-vaccination titers against B/Austria/1359417/2021(Victoria lineage), which was first included in the vaccine formulation in 2022/23. High pre-vaccination titers likely stem from cross-reactivity [32–34], or previous circulation of similar strains in Asia, including triple deletions in 2018 [35], and additional mutations detected at A127T, P144L, and K203R in 2020 [36].

We found that A/Cambodia/e0826360/2020(H3N2) and A/Darwin/9/2021(H3N2) had much lower titer levels at days 14 and 180 (Supplementary Table 3) as well as boosts (Figure 2) compared to all other vaccine strains, including A/Hong Kong/4801/2014, the other H3N2 strain tested. Although H3N2 strains generally have lower vaccine effectiveness compared to H1N1 strains [37], the lower A/Cambodia/e0826360/2020(H3N2) titers observed may reflect differences in receptor avidity rather than true differences in immunity [14]. In addition, it has been shown that antibodies elicited in response to the egg-adapted A/Hong Kong/4801/2014 vaccine strain poorly neutralize glycosylated clade 3C.2a viruses that have been circulating since 2014/15 [38]. As such, the poor responses seen in the H3N2 strains may be influenced by the lack of a new glycosylation site at HA antigenic site B in the vaccine strains.

Our findings on waning antibody levels in repeat vaccinees differ from those previously reported by Zelner et al [39] and Hodgson et al [10]. We found evidence of slower antibody waning in the repeat vaccinees, who had lower post-vaccination titers in the boosting analysis (Figure 2) and the estimated intercepts of the waning models (Figure 4), but reached similar antibody levels at day 180. Importantly, there was no influenza circulation in Hong Kong during this period [12], so the earlier infection of vulnerable participants, which would leave behind a less susceptible population, was not a factor. Therefore, antibody trajectories were shaped solely by vaccination. In contrast, Zelner et al. reported faster waning and predicted reduced 6-month levels with repeated same-strain vaccination [39]. Hodgson et al. found persistent differences at 6 months post-vaccination [10]. We are unable to reconcile these differences, but analysis of pooled data with a variety of methods might provide insights.

Our study was limited by the size of the subcohort of repeat vaccinees analyzed. Although EPI-HK has enrolled over 2500 participants, the timing of serum sample collections limited the participants we could include within this study and may be insufficient to conclusively determine the effects of repeated seasonal influenza vaccination. Due to the nature of the study design, we were unable to collect day 0 and day 30 serum samples to study antibody boosting. Instead, we used a range up to 60 days after vaccination, which may result in an underestimation of antibody boosting and fold-change. However, no significant differences between vaccination history groups were observed in the timing of post-vaccination serum collection (Supplementary Figure 1). Similarly, most participants only had 2 time points for modeling antibody waning, which limited our ability to fit more complex individual trajectories. However, the range of time points during a period with no influenza circulation is also a strength of the study, allowing us to fit a curve to data collected over a longer time period where antibody dynamics were only perturbed by vaccinations. Vaccination history in the prior 6 years was collected by self-report and may be misreported although our analyses did identify repeat vaccination effects as expected. We also did not collect data on other immune parameters such as cellular immunity that may be related to vaccination responses or use functional assays such as microneutralization (MN) which may also provide relevant insights into potential vaccine effectiveness. Nevertheless, HAI is the established correlate of protection for vaccine efficacy and studies have shown strong correlations between HAI and MN antibody levels [40, 41].

In conclusion, we observed some evidence of attenuation in HAI titers in repeat vaccinees, with significant differences in MFRs for 2 H3N2 strains after accounting for ceiling effects. However, despite slower waning rates and lower HAI titers at 14–60 days post-vaccination in the high vaccination uptake group, antibody titer levels were similar at 6 months post-vaccination.

## Supplementary Material

jiag051_Supplementary_Data

## Supplementary Data

Supplementary materials are available at *The Journal of Infectious Diseases* online (http://jid.oxfordjournals.org/). Supplementary materials consist of data provided by the author that are published to benefit the reader. The posted materials are not copyedited. The contents of all supplementary data are the sole responsibility of the authors. Questions or messages regarding errors should be addressed to the author.
