# Supplementary material for: Repeat Influenza Vaccination Effects in 2021/22 and 2022/23 in a Community-Based Cohort in Hong Kong
Source: J Infect Dis. 2026 Jan 28;233(6):e1364–73. doi: 10.1093/infdis/jiag051 (PMC13271417; doi:10.1093/infdis/jiag051)
Supplement: jiag051_Supplementary_Data [file jiag051_supplementary_data.pdf]

APPENDIX

Repeat influenza vaccination effects in 2021/22 and 2022/23 in a community-based cohort in Hong Kong

Appendix Figure 1. Timing of return visit post-vaccination by vaccination history in 2021/22 and 2022/23. The low R^2 values indicate very weak correlations.

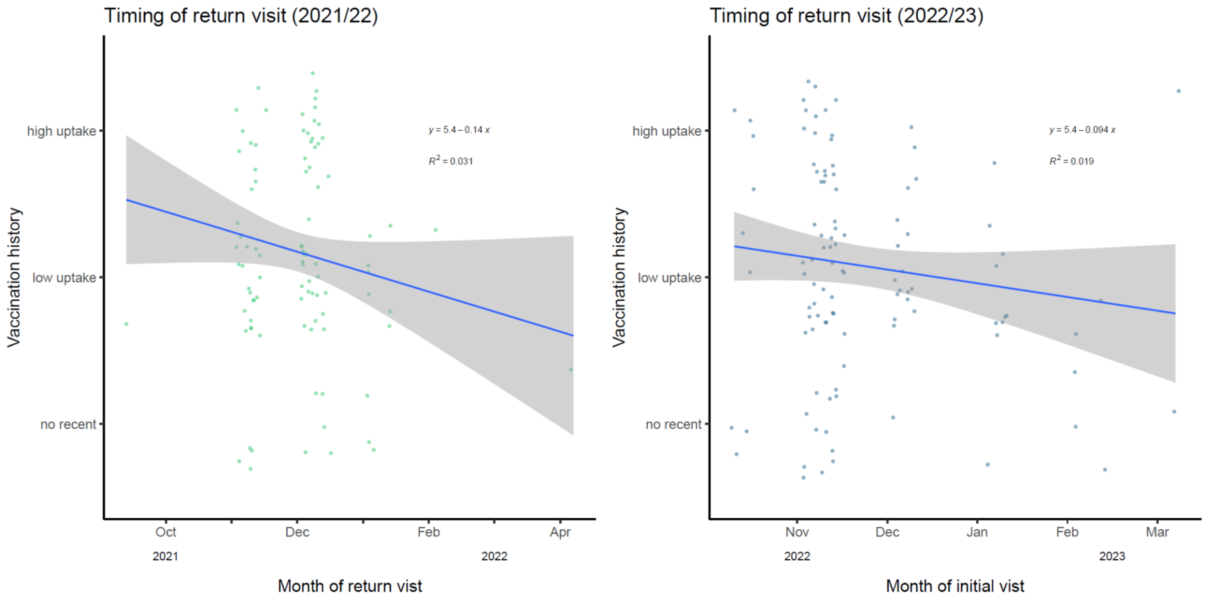

Appendix Table 1. Vaccine strains tested, with specific clades and seasons included in the northern hemisphere vaccine. Vaccine strains 2021/22 and 2022/23 are highlighted in bold.

**Type/Subtype**	**Strain**	**Clade**	**Northern hemisphere season**
A(H1N1)	A/Brisbane/2/2018	6B.1A.1	2019/20
**A(H1N1)**	**A/Victoria/2570/2019**	6B.1A5A+156K	**2021/22 - 2022/23**
A(H3N2)	A/Hong Kong/4801/2014	3C.2a	2016/17 - 2017/18
**A(H3N2)**	**A/Cambodia/e0826360/2020**	3C.2a1b.2a.1	**2021/22**
**A(H3N2)**	**A/Darwin/9/2021**	2a	**2022/23**
**B/Victoria**	**B/Washington/02/2019**	V1A.3	**2020/21 - 2021/22**
**B/Victoria**	**B/Austria/1359417/2021**	V1A.3a.2	**2022/23**
**B/Yamagata**	**B/Phuket/3073/2013**	Yamagata lineage	**2015/16 - 2022/23**

Appendix Table 2. Akaike Information Criterion (AIC) scores for three different antibody waning model formulations for tested influenza strains.

**Strain**	**Biphasic**	**Exponential**	**Power**
A/Brisbane/2/2018 (H1N1)	1522.48	1255.53	1228.06
A/Victoria/2570/2019 (H1N1)	1435.45	1219.13	1192.55
A/Hong Kong/4801/2014 (H3N2)	1441.09	1221.58	1200.38
A/Cambodia/e0826360/2020 (H3N2)	1274.56	1122.72	1097.08
A/Darwin/9/2021 (H3N2)	1230.61	1078.52	1060.77
B/Washington/02/2019 (Victoria lineage)	1385.70	1223.65	1203.98
B/Austria/1359417/2021 (Victoria lineage)	1127.09	1038.22	1019.11
B/Phuket/3073/2013 (Yamagata lineage)	1398.01	1179.96	1162.03

Appendix Table 3: Estimated hemagglutination inhibition (HAI) titers at 14 and 180 days post-vaccination summarised by vaccination history and vaccination strain with 95% CI.

**Strain**	**No vaccination for six prior years**	**Low uptake (1-2 vaccinations in prior six years)**	**High uptake (≥3 vaccinations in prior six years)**
Day 14 HAI titers			
A/Brisbane/2/2018 (H1N1)	138 *(137, 139)*	140 *(139, 141)*	111 *(110, 111)*
A/Victoria/2570/2019 (H1N1)	159 *(158, 160)*	118 *(118, 119)*	87 *(86, 88)*
A/Hong Kong/4801/2014 (H3N2)	96 *(95, 98)*	103 *(102, 104)*	95 *(94, 96)*
A/Cambodia/e0826360/ 2020 (H3N2)	44 *(43, 46)*	42 *(41, 43)*	26 *(25, 27)*
A/Darwin/9/2021 (H3N2)	35 *(33, 36)*	35 *(34, 35)*	20 *(19, 20)*
B/Washington/02/2019 (Victoria lineage)	114 *(113, 115)*	113 *(112, 114)*	76 *(75, 76)*
B/Austria/1359417/2021 (Victoria lineage)	488 *(487, 489)*	371 *(371, 372)*	253 *(252, 253)*
B/Phuket/3073/2013 (Yamagata lineage)	120 *(119, 121)*	125 *(124, 126)*	78 *(77, 79)*
Day 180 HAI titers			

A/Brisbane/2/2018 (H1N1)	60 *(58, 61)*	62 *(61, 63)*	56 *(55, 57)*
A/Victoria/2570/2019 (H1N1)	61 *(59, 62)*	53 *(52, 54)*	43 *(42, 44)*
A/Hong Kong/4801/2014 (H3N2)	51 *(50, 53)*	56 *(55, 56)*	61 *(60, 62)*
A/Cambodia/e0826360/2020 (H3N2)	26 *(25, 28)*	24 *(23, 24)*	19 *(18, 19)*
A/Darwin/9/2021 (H3N2)	24 *(22, 25)*	26 *(25, 27)*	20 *(20, 21)*
B/Washington/02/2019 (Victoria lineage)	70 *(68, 71)*	80 *(79, 81)*	51 *(50, 52)*
B/Austria/1359417/2021 (Victoria lineage)	328 *(327, 329)*	299 *(299, 300)*	234 *(234, 235)*
B/Phuket/3073/2013 (Yamagata lineage)	73 *(72, 74)*	80 *(79, 81)*	81 *(80, 82)*
